# Activity Augmentation of Amphioxus Peptidoglycan Recognition Protein BbtPGRP3 via Fusion with a Chitin Binding Domain

**DOI:** 10.1371/journal.pone.0140953

**Published:** 2015-10-19

**Authors:** Wen-Jie Wang, Wang Cheng, Ming Luo, Qingyu Yan, Hong-Mei Yu, Qiong Li, Dong-Dong Cao, Shengfeng Huang, Anlong Xu, Roy A. Mariuzza, Yuxing Chen, Cong-Zhao Zhou

**Affiliations:** 1 Hefei National Laboratory for Physical Sciences at Microscale and the Innovation Center for Cell Signaling Network, School of Life Sciences, University of Science and Technology of China, Hefei, Anhui, China; 2 State Key Laboratory of Biocontrol, Guangdong Key Laboratory of Pharmaceutical Functional Genes, School of Life Sciences, Sun Yat-sen University, Guangzhou, China; 3 University of Maryland Institute for Bioscience and Biotechnology Research, W. M. Keck Laboratory for Structural Biology, Rockville, Maryland, United States of America; 4 Department of Cell Biology and Molecular Genetics, University of Maryland, College Park, Maryland, United States of America; Institut Pasteur Paris, FRANCE

## Abstract

Peptidoglycan recognition proteins (PGRPs), which have been identified in most animals, are pattern recognition molecules that involve antimicrobial defense. Resulting from extraordinary expansion of innate immune genes, the amphioxus encodes many PGRPs of diverse functions. For instance, three isoforms of PGRP encoded by *Branchiostoma belcheri tsingtauense*, termed BbtPGRP1~3, are fused with a chitin binding domain (CBD) at the N-terminus. Here we report the 2.7 Å crystal structure of BbtPGRP3, revealing an overall structure of an N-terminal hevein-like CBD followed by a catalytic PGRP domain. Activity assays combined with site-directed mutagenesis indicated that the individual PGRP domain exhibits amidase activity towards both DAP-type and Lys-type peptidoglycans (PGNs), the former of which is favored. The N-terminal CBD not only has the chitin-binding activity, but also enables BbtPGRP3 to gain a five-fold increase of amidase activity towards the Lys-type PGNs, leading to a significantly broadened substrate spectrum. Together, we propose that modular evolution via domain shuffling combined with gene horizontal transfer makes BbtPGRP1~3 novel PGRPs of augmented catalytic activity and broad recognition spectrum.

## Introduction

The innate immune system is the first line of defense for vertebrates to fight against invading microorganisms and the only defense system for invertebrates and plants [1, 2]. This system provides recognition of relatively invariable exogenous products of microbial metabolism, termed pathogen-associated molecular patterns (PAMPs), through a limited number of germ line-encoded pattern-recognition receptors (PRRs) [3, 4]. One representative example of PAMPs is peptidoglycan (PGN), an essential and unique cell-wall component of both Gram-positive and negative bacteria. PGNs are normally recognized, and/or hydrolyzed (amidases *EC 3*.*5*.*1*.*28*) in some cases, by a class of PRRs, termed peptidoglycan recognition proteins (PGRPs). Both invertebrates and vertebrates encode a large amount of PGRPs of varying functions [5]. For example, the insects have up to 19 PGRPs, which function as either sensors or effectors. Sensor PGRPs recognize pathogens and activate innate signaling pathways including Toll (*Drosophila* PGRP-SA, PGRP-SD, and PGRP-SC1) [6–8], Imd (*Drosophila* PGRP-LC) [9], and prophenol-oxidase cascade (silkworm PGRP-S) [10, 11], whereas effector PGRPs, such as *Drosophila* PGRP-SC1, PGRP-LB, and PGRP-SB1, have either direct bactericidal or amidase activities [12–14]. In contrast, mammals possess four PGRPs: PGLYRP-1~4, all of which serve as effectors [15–17].

As the proximate ancestor of vertebrates, the cephalochordate amphioxus harbors 17–18 PGRP genes, however, none of which has been reliably clustered with insect or mammalian PGRPs [18]. Bioinformatics analyses indicated that all amphioxus PGRPs have potential amidase activity; thus they should all function as effector or catalytic PGRPs [19]. The catalytic PGRPs possess the amidase activity that hydrolyzes the amide bond between the MurNAc and L-alanine moieties of PGN, via a coordinated Zn^2+^ ion [20]. The active site consists of two histidines in addition to a tyrosine and a cysteine residue, which determines the gain or loss of amidase activity [21]. In addition, the amidase activity is necessary for PGRPs to play a scavenger role [13, 22].

Three isoforms of PGRP genes BbtPGRP1~3 from the amphioxus *Branchiostoma belcheri tsingtauense* have been deposited in GenBank under the accession numbers of AEU03853.1, AEU03854.1 and KR136228. The encoded hypothetical proteins are featured with a chitin-binding domain (CBD) fused at the N-terminus. CBDs are widespread across the animal and plant kingdoms [23, 24] and exhibit antifungal and/or antimicrobial activity [25–27], through specific binding to carbohydrates [28].

Here we report the crystal structure of BbtPGRP3 at 2.7 Å resolution, showing an N-terminal hevein-like CBD followed by a classic PGRP domain at the C-terminus. Structural analyses combined with a series of binding assays and site-directed mutagenesis indicated that the two key residues Trp53 and Trp55 in CBD are involved in chitin recognition, and the PGRP domain exhibits a higher *in vitro* hydrolytic activity towards the DAP-type PGNs compared to the Lys-type. Moreover, fusion with the CBD significantly augments the amidase activity towards the Lys-type PGNs, leading to a broader spectrum to recognize both Gram-positive and negative bacteria. All together, the modular evolution via gene horizontal transfer and domain shuffling results in a unique domain organization of BbtPGRP3, which has a higher amidase activity and a broader spectrum against invading bacteria.

## Results and Discussion

### Overall structure of BbtPGRP3

**Fig 1:**
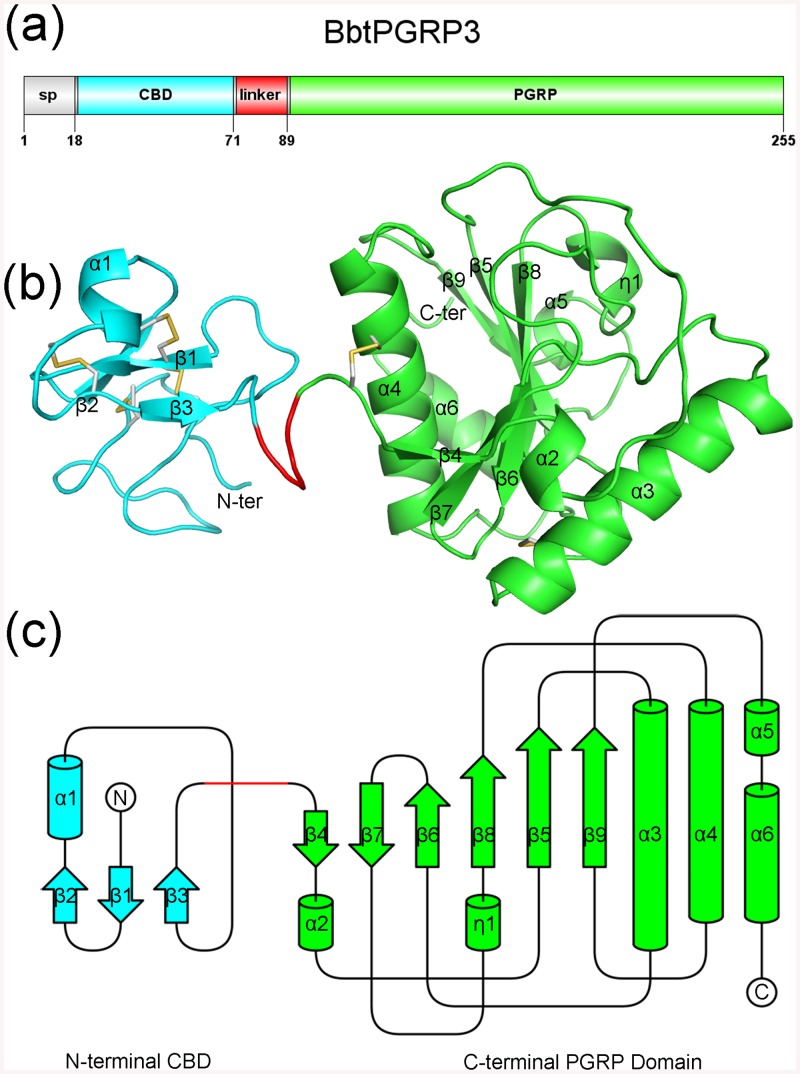
Overall structure of BbtPGRP3. (**a**) Domain organization of BbtPGRP3. (**b**) Structure of BbtPGRP3. The secondary structure elements are numbered in the order of appearance in the primary sequence. The N and C-termini are indicated. (**c**) Topology diagram of BbtPGRP3. The N- and C-terminal domains are shown in cyan and green, respectively. The linker is shown in red.

The full-length BbtPGRP3 consists of an N-terminal signal peptide (Met1−Ala18), followed by a CBD (Gln19−Tyr71) and a C-terminal PGRP domain (Thr90−Gly255), which are connected via an 18-residue linker from residues Ser72 to Gly89 (Fig 1a). The refolded protein of BbtPGRP3, which were overexpressed in *Escherichia coli* as the insoluble form, were initially applied to crystallization; however, the final model indicated that the crystal diffracting at 2.80 Å resolution (PDB code 4ZXM) contains only the C-terminal PGRP domain (Thr90−Val254). Moreover, the full-length protein expressed in Sf9 insect cells produced crystals of poor quality, which could not be optimized to an acceptable resolution. Thus, we truncated the inter-domain linker from 18 to 6 residues, and finally obtained the crystal at a diffraction resolution of 2.70 Å (PDB code 4Z8I).

Each asymmetric unit of the crystal contains one molecule of BbtPGRP3 (covering residues Gln19−Gly74 and Ser87−Gly254). It is composed of an N-terminal CBD (Gln19−Tyr71) and a C-terminal PGRP domain (Thr90−Val254) connected by a truncated linker (Ser72−Gly74, Ser87−Gly89) (Fig 1b). The topology diagram using the programs PDBsum (http://www.ebi.ac.uk/thornton-srv/databases/pdbsum/) and TopDraw [29] further confirmed that BbtPGRP3 consists of two distinct domains (Fig 1c). Notably, truncation of the linker from 18 to 6 residues did not lead to significant change of the amidase activity; thus only the wild-type BbtPGRP3 was applied to all following activity assays and comparisons.

### The N-terminal CBD

**Fig 2:**
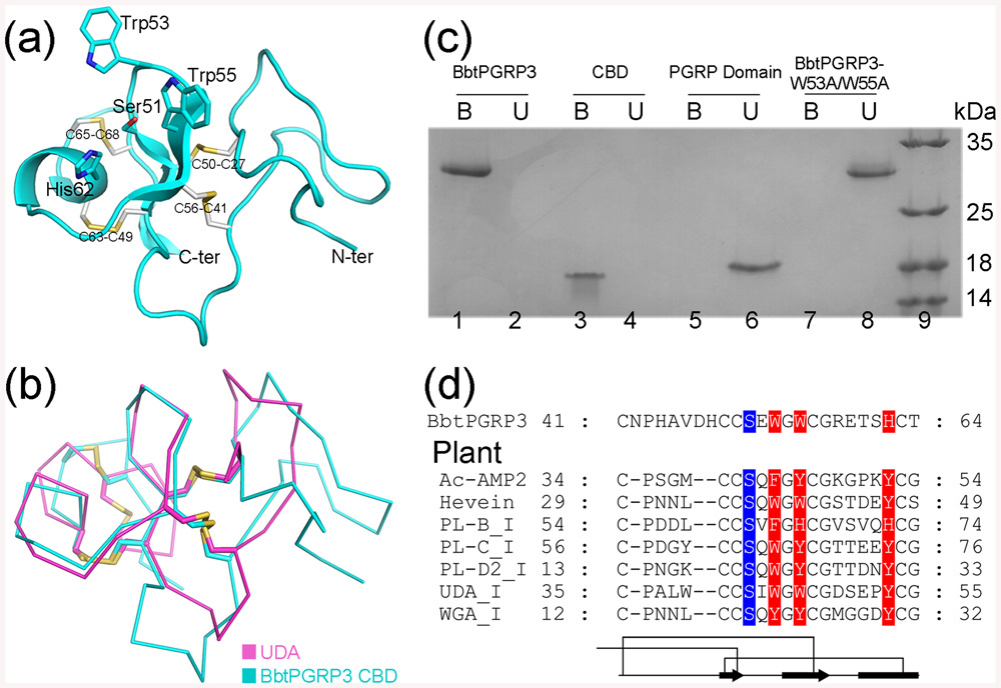
The N-terminal CBD of BbtPGRP3. (**a**) Cartoon representation of the BbtPGRP3 N-terminal CBD. The side chains of the conserved cysteine and putative functional residues are shown, with the conserved disulfide bridges colored in yellow. (**b**) Superposition of BbtPGRP3 CBD against UDA (PDB 1ENM). The CBD and UDA are colored in cyan and magenta, respectively. (**c**) The chitin-binding activity. Purified BbtPGRP3, CBD, PGRP domain and BbtPGRP3-W53A/W55A were incubated with insoluble chitin, respectively. Protein remaining in the supernatant (Unbound) and associated with the pellet (Bound) were analyzed by SDS-PAGE and Coomassie staining. (**d**) 3D structure-based sequence alignment of several chitin-binding proteins in plants against the Cys41–Thr64 region of BbtPGRP3. The proteins include *Amaranthus caudatus* antimicrobial protein 2 (Ac-AMP2, PDB code 1MMC), hevein from rubber tree (Hevein, PDB code 1T0W), *Phytolacca americana* Lectin-B (PL-B_I, UniProtKB accession number Q9AVB0.1), *Phytolacca americana* Lectin-C (PL-C_I, PDB code 1ULK), *Phytolacca americana* Lectin-D (PL-D_I, PDB code 1ULM), *Urtica dioica* agglutinin (UDA_I, PDB code 1IQB), and wheat germ agglutinin (WGA_I, PDB code 1WGC). The chitin-binding residues are aligned. The polar and hydrophobic residues are highlighted in blue and red, respectively.

The CBD starts with a long N-terminal loop (Gln19−Glu40), followed by the core structure of a three-stranded antiparallel β-sheet (β1~3) and a helix α1 (Fig 2a). It displays a 30-residue structural motif, which is organized around a four-disulfide core, similar to member of the hevein-like agglutinin (lectin) domain family [30, 31], in agreement with the prediction of the SUPERFAMILY web server (http://supfam.org/SUPERFAMILY/index.html). Structural comparison using the Dali server [32] gave 59 hits for 35 unique proteins with a Z-score ranging from 5.2 to 2.0, all of which are members of hevein-like agglutinin (lectin) domain family of the plant lectins/antimicrobial peptides superfamily. The top hit is an agglutinin isolectin I from *Urtica dioica* (PDB code 1IQB; Z-score of 5.2, RMSD of 2.2 Å over 44 Cα atoms) [33], followed by *Urtica dioica* agglutinin UDA (code 1ENM) [34] and *Phytolacca americana* Lectin-C (code 1ULK) [35]. The other hits include *Phytolacca americana* Lectin-D2 (code 1UHA) [36], wheat germ agglutinin isolectin from *Triticum aestivum* (code 1WGC) [37], *Triticum aestivum* agglutinin isolectin 1 (code 2UVO) [38], class I chitinase from *Oryza sativa Japonica Group* (code 2DKV) [39], and *Hevea brasiliensis subsp*. *brasiliensis* Class I chitinase (code 4MPI) [40].

The CBD of BbtPGRP3 adopts an overall structure remarkably similar to the well-characterized *U*. *dioica* UDA [34]. Besides, the four disulfide bridges (Cys27-Cys50, Cys41-Cys56, Cys49-Cys63, and Cys65-Cys68) that stabilize the BbtPGRP3 CBD also resemble the counterparts in UDA (Fig 2b). The aromatic side chains of Trp44, Trp46, and Tyr53 of UDA are known to specifically bind to a chitin-derived oligosaccharide, which is further stabilized by two hydrogen bonds with Ser42 and Tyr53 [34]. The residues of Ser51, Trp53, Trp55 and His62 of BbtPGRP3 CBD correspond to the residues of Ser42, Trp44, Trp46, and Tyr53 of UDA. All the four putative chitin-binding residues of the CBD of BbtPGRP3 could be superimposed to those in the structure of UDA complexed with a trisaccharide. Binding assays revealed that the entire amount of the full-length BbtPGRP3 or the N-terminal CBD binds to chitin beads in our experimental conditions (Fig 2c). In contrast, neither the individual C-terminal PGRP domain nor the double mutant BbtPGRP3-W53A/W55A is able to bind to the chitin beads (Fig 2c).

Structure-based sequence alignment of the core chitin-binding motif (Cys41−Thr64) of the CBD against the plant chitin-binding proteins strongly indicated the conservation of this motif among plants and amphioxus (Fig 2d). The functional residues in plant chitin-binding proteins include three aromatic residues and a polar residue, whereas the invertebrate chitin-binding proteins possess two hydrophobic residues and a polar residue [24]. It suggests that BbtPGRP3 CBD shows a relatively high similarity to the plant chitin-binding proteins from the sequence and structural points of view.

It was hypothesized that chitin-binding proteins of invertebrates and plants are correlated by a convergent-evolution process [24, 41], as seen from the conserved structural motifs of chitin-binding proteins in invertebrates and plants [42]. The amphioxus genome encodes more than 250 hypothetical CBDs, most of which are of unknown function [43]. These CBDs could be classified into three functionally distinct groups: the chitinases, the plant-like and invertebrate-like CBDs [19]. Besides BbtPGRP3, BbtPGRP1/2 also contain a plant-like CBD, which has not been found in any other chordates, except for amphioxus, strongly suggesting a horizontal gene transfer from plants. In contrast, the *Branchiostoma floridae* variable region-containing chitin-binding proteins (BfVCBPs) [43] possess CBDs that belong to the invertebrate chitin-binding protein superfamily according to the prediction of SUPERFAMILY web server [30], indicating amphioxus CBDs were derived from diverse evolutionary origins.

### The C-terminal PGRP domain

**Fig 3:**
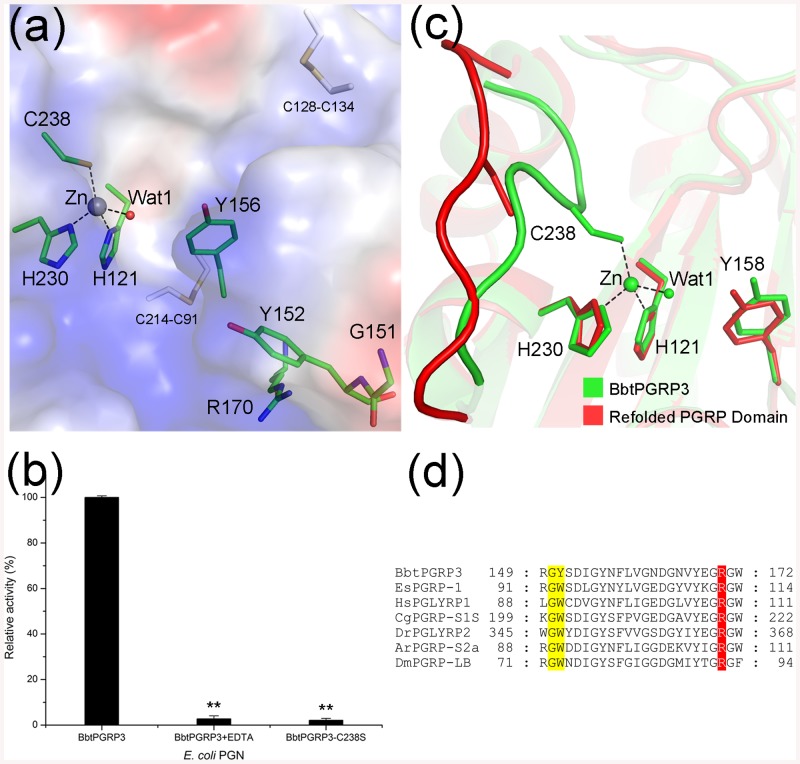
The C-terminal amidase domain of BbtPGRP3. (**a**) Surface representation of the BbtPGRP3 C-terminal PGRP domain. The zinc ion and its coordinating water molecule are displayed as lightblue and red spheres, respectively. The active-site residues and putative residues related to PGN-binding specificity are shown as sticks and colored in green. (**b**) Reactions containing RBB-labeled *E*. *coli* PGNs were incubated with 50 nM wild-type BbtPGRP3, EDTA-treated BbtPGRP3 and BbtPGRP3-C238S, respectively. Undigested PGNs were pelleted by centrifugation and the absorbance of the supernatants at 595 nm were determined. Results of activity are expressed as the percentage to the wild-type BbtPGRP3. The values are expressed as mean values ± SD of three independent experiments. All data were analyzed by *t*-test, and the statistical significance of the differences between experimental and a control group is shown with asterisks (**P<0.005). (**c**) Superposition of PGRP domain from full-length BbtPGRP3 against the refolded PGRP domain. The full-length BbtPGRP3 and refolded PGRP domain are colored in green and red, respectively. The zinc ion and its coordinating water molecule are displayed as spheres and the active-site residues are shown as sticks. (**d**) Multiple-sequence alignment of specificity-determining residues of PGRPs. Residues corresponding to Gly150/Tyr151 and Arg170 of BbtPGRP3 are highlighted in yellow and red, respectively. The sequences are (NCBI accession numbers codes in parentheses) *Branchiostoma belcheri tsingtauense* BbtPGRP3 (KR136228), *Asterias rubens* ArPGRP-S2a (ABB04460.1), *Crassostrea gigas* CgPGRP-S1S (BAG31896.1), *Drosophila melanogaster* DmPGRP-LB (NP_650079.1), *Danio rerio* DrPGLYRP2 (NP_001038631.1), *Euprymna scolopes* EsPGRP-1 (AAY27973.1) and *Homo sapiens* HsPGLYRP1 (NP_005082.1).

The PGRP domain shows a typical PGN-binding domain scaffold, in which six β-strands (β4~9) compose a central β-sheet surrounded by five α-helices (α2~6) and a short 3_10_ helix η1 (Fig 1b and 1c). The two disulfide bonds (Cys91-Cys214 and Cys128-Cys134) further stabilize the PGRP domain. At the center of the solvent-exposed valley, a zinc ion is tetrahedrally coordinated by the nitrogen atoms of residues His121, His230, the sulfur atom of Cys238, and a water molecule Wat1 (Fig 3a). Wat1 is further stabilized by the Oη atoms of the catalytic residue Tyr156, which have been proposed to facilitate a water-mediated nucleophilic attack on the lactyl-amide bond and stabilize the reaction intermediate, respectively [20]. Either single mutation of Cys238 or treatment of BbtPGRP3 with EDTA completely abolished the amidase activity towards *E*. *coli* PGNs (Fig 3b), indicating the critical role of the zinc ion and coordinating residues for catalysis. Structural comparison revealed that the PGRP domain in the full-length BbtPGRP3 structure (PDB code 4Z8I) and the refolded PGRP domain without zinc (PDB code 4ZXM) share an almost identical overall structure with an RMSD of 0.544 Å over 162 Cα atoms; however, binding of the zinc ion makes a significant induced fit of the loop region from Gly234 to Asn241 that contains the coordinating residue Cys238 (Fig 3c).

**Fig 4:**
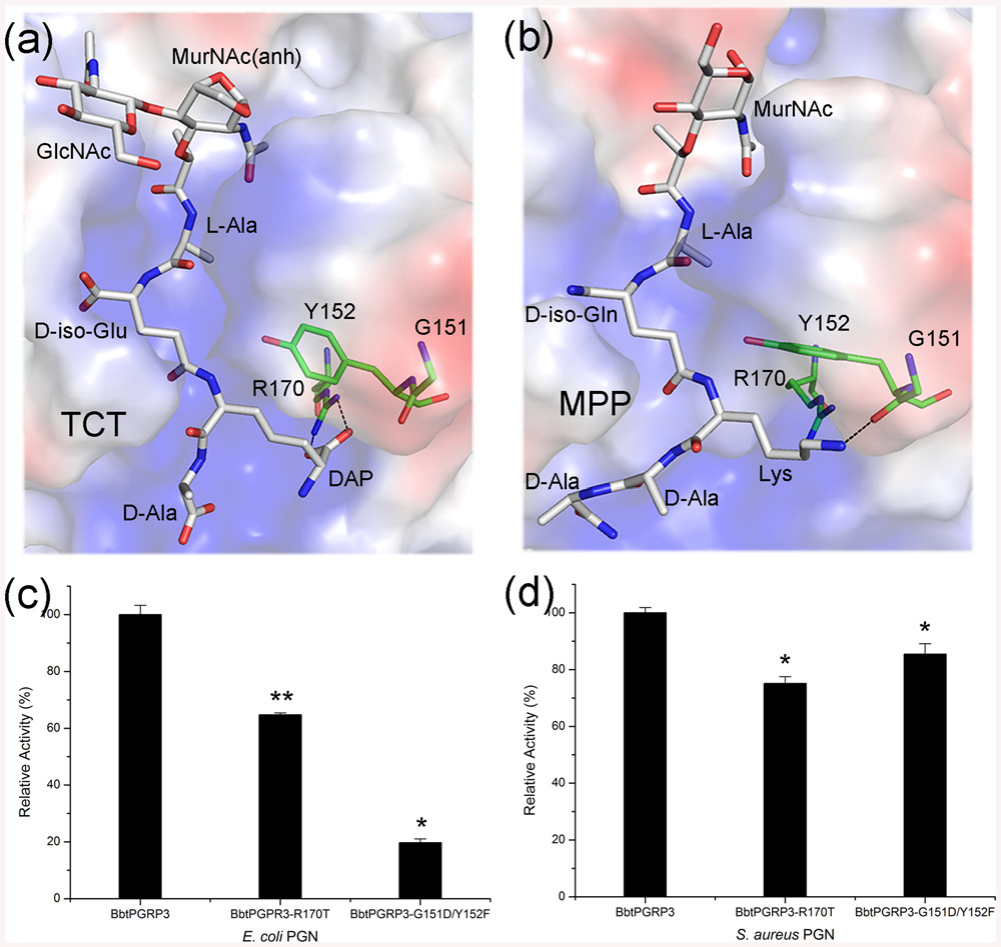
The G/Y/R motif and amidase activity. Docking models of (**a**) TCT or (**b**) MPP binding to the PGRP domain. TCT and MPP are colored in white, whereas the interacting residues are colored in green. Reactions containing RBB-labeled (**c**) *E*. *coli* PGNs or (**d**) *S*. *aureus* PGNs were incubated with wild-type BbtPGRP3, the R170T and G151D/Y152F mutants, respectively. Undigested PGNs were pelleted by centrifugation and the absorbance of the supernatants at 595 nm were determined. Results of activity are expressed as the percentage to the wild-type. The values are expressed as mean values ± SD of three independent experiments. All data were analyzed by *t*-test, and the statistical significance of the differences between experimental and a control group is shown with asterisks (*P<0.01, **P<0.005).

On the basis of PGN-binding specificity, PGRPs have been classified into four groups. PGRPs in Group I and III recognize the Lys-type PGNs, whereas those in Group II prefer the DAP-type [44, 45]. Multiple-sequence alignment showed that BbtPGRP3 possess a highly conserved Gly-Tyr motif (Gly150-Tyr151) and an arginine residue Arg170 (Fig 3d), which are featured in the Group II PGRPs. Using HADDOCK server [46], we docked tracheal cytotoxin (TCT, GlcNAc-MurNAc(1,6-anhydro)-_L_-Ala-γ-_D_-Glu-*meso*-Dap-_D_-Ala) and muramyl pentapeptide (MPP, MurNAc-_L_-Ala-_D_-isoGln-_L_-Lys-_D_-Ala-_D_-Ala), which represent the DAP-type and Lys-type PGNs respectively, to the active-site valley of PGRP domain (Fig 4a and 4b). The simulated binding patterns of TCT and MPP are similar to those previously reported structures of DmPGRP-LE-TCT [47] and HsPGlyRP3-MPP [48] complexes, respectively. The side-chain carboxylate of DAP forms a bidentate salt bridge with the guanidinium group of the conserved Arg170 docked TCT-complexed model (Fig 4a), whereas residues Gly150 and Tyr151 interact with the side chain of L-Lys via van der Waals interactions in the MPP-complexed model (Fig 4b). Moreover, structural comparison suggested that the complementary electropotential between DAP and Arg170 makes DAP-type PGN a favored substrate. Due to the unavailability of TCT, MPP was applied to HPLC assays and the results revealed that BbtPGRP3 is indeed an amidase that hydrolyze MPP to produce the pentapeptide (S1 Fig). Moreover, the *in vitro* binding assays further confirmed that the DAP-type PGNs are indeed preferred by BbtPGRP3 (S2 Fig).

To further prove the role of the Gly-Tyr-Arg motif in the recognition of DAP-type PGNs, a single mutant BbtPGRP3-R170T and a double mutant BbtPGRP3-G151D/Y152F were applied to the *in vitro* hydrolytic activity assays. As shown in Fig 4c, the single mutation and double mutation led to an approximately 35% and 80% decrease in activity towards purified *E*. *coli* (DAP-type) PGNs. In contrast, the activities of the two mutants towards the *Staphylococcus aureus* (Lys-type) PGNs were less significantly reduced (Fig 4d). These results were consistent with our previously hypothesis [45]. Mutation of residues Gly-Tyr to Asp-Phe would result in a change of PGN-binding specificity. The side chain of Asp151 would create steric clashes with DAP and lead to a decreased affinity. However, As the Gly151 residue interacts with Lys via its main-chain oxygen, either Gly-Tyr or Asp-Phe motif could bind to the Lys residue of MPP. In addition, we compared the hydrolysis rates of BbtPGRP3 to both types of PGNs, and the results demonstrated that the DAP-type PGNs are favorite substrates (S3 Fig).

### Inter-domain cooperativity and putative evolution

**Fig 5:**
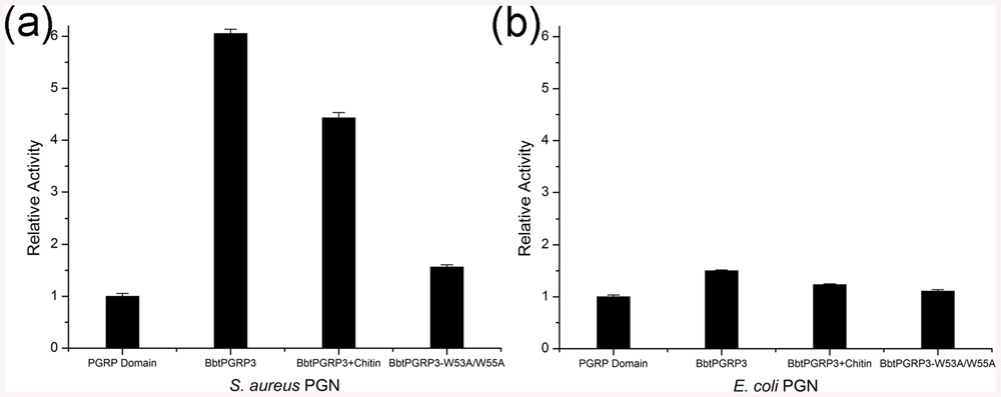
Cooperation of the N- and C-terminal domains of BbtPGRP3. Reactions containing RBB-labeled (**a**) *S*. *aureus* PGNs or (**b**) *E*. *coli* PGNs were incubated with 50 nM PGRP domain, BbtPGRP3 in the absence or presence of chitin and BbtPGRP3-W53A/W55A, respectively. Undigested PGNs were pelleted by centrifugation and the absorbance of the supernatants at 595 nm were determined. Results of activity are expressed as the folds to that of individual PGRP domain. The values are expressed as mean values ± SD of three independent experiments.

To further investigate the putative cooperation between the two domains, we compared the *in vitro* hydrolytic activities of the individual PGRP domain, the wild-type BbtPGRP3 and the double mutant BbtPGRP3-W53A/W55A, in the absence or presence of chitin. Compared to the individual PGRP domain, fusion of the CBD resulted in an approximately 500% augmentation of activity towards *S*. *aureus* PGNs (Fig 5a), compared to a 50% increase towards *E*. *coli* PGNs (Fig 5b). It indicated that CBD contributes to the hydrolytic activity towards PGNs (especially the Lys-type) of the PGRP domain. The presence of chitin led to an approximately 30% decrease in activity towards *S*. *aureus* PGNs as compared to the wild-type (Fig 5a), suggesting that the enhancement of amidase activity is mediated by the chitin-binding site of the CBD. Moreover, double mutations of the two chitin-binding residues Trp53 and Trp55 to Ala resulted in a 75% decrease of activity towards *S*. *aureus* PGNs, which is almost at the same level to that of the individual PGRP domain (Fig 5a). In contrast, the presence of chitin or the mutation of W53/W55 only led to a slight decrease (approximately 20% to 30%) of activity towards *E*. *coli* PGNs (Fig 5b). Taken together, the cooperation between the CBD and PGRP domain significantly augments the amidase activity towards Lys-type PGNs, thus broadens the recognition spectrum of BbtPGRP3.

This novel domain combination of a CBD fusion with a PGRP domain has been only found in amphioxus, which might result from domain shuffling when a great expansion of amphioxus immune gene repertoire occurred [18]. Moreover, both plant-like and invertebrate-like CBDs have been found from amphioxus, indicating that they more likely originated in “modular evolution” [49–51], which undergoes horizontal gene transfer, rather than “convergent evolution”. All together, fusion with a CBD makes BbtPGRP3 novel PGRP of augmented amidase activity and broadened spectrum against invading bacteria.

## Materials and Methods

### Plasmid Construction and Preparation of BbtPGRP3

The cDNA coding BbtPGRP3 (Gln19−Gly255) was cloned into a 2BT vector (QB3 MacroLab) by a standard Ligation Independent Cloning (LIC) method, and expressed as inclusion bodies in BL21 (DE3) *E*. *coli* cells (Novagen). Bacteria were grown at 37°C in LB medium to an absorbance of 0.6–0.8 at 600 nm, and induced with 1 mM IPTG. After incubation for 4 h, the bacteria were harvested by centrifugation and resuspended in 50 mM Tris-HCl, pH 8.5, containing 150 mM NaCl and 2 mM EDTA; cells were disrupted by sonication. Inclusion bodies were washed with 50 mM Tris-HCl, pH 8.5, 150 mM NaCl and 1% (*v/v*) Triton X-100 for 3 times, washed with 50 mM Tris-HCl, pH 8.5, 150 mM NaCl for 3 times, and then solubilized in 8 M urea and 100 mM Tris-HCl, pH 8.5. For *in vitro* folding, inclusion bodies were diluted to a final concentration of 10 mg/l into 100 mM Tris-HCl, pH 8.5, 0.8 M arginine, 2 mM EDTA, 3 mM reduced glutathione and 0.3 mM oxidized glutathione. After 5 days at 4°C, the folding mixture was concentrated, dialyzed against 20 mM Tris-HCl, pH 8.5, and applied to a Superdex 75 10/300 GL (GE Healthcare) column. Fractions containing the target protein were collected and concentrated to 10 mg/ml for crystallization [52].

However, the N-terminal CBD of refolded BbtPGRP3 was degraded during the crystallization process as proved by SDS-PAGE, the coding regions of BbtPGRP3 (Gln19−Gly255), BbtPGRP3 CBD (Gln19−Tyr71), BbtPGRP3 PGRP domain (Thr90−Gly255) and BbtPGRP3 (Gln19−Gly74 and Ser87−Gly255, only used in crystallization) were cloned into the pAcGP67-B vector (BD Biosciences) in frame with the baculovirus gp67 signal sequence. Also mutation versions of full-length BbtPGRP3, BbtPGRP3-R170T, BbtPGRP3-G151D/Y152F, BbtPGRP3-C238S, and BbtPGRP3-W53A/W55A were constructed. Sf9 insect cells (Invitrogen) were contransfected with one of the constructs and BestBac DNA (Expression Systems) to produce a recombinant baculovirus expressing protein. Virus stocks were amplified with three sequential infections of Sf9 cells. The resulting His-tagged hybrids were expressed in Sf9 cells (Invitrogen) and purified by Ni Sepharose excel affinity chromatography (GE Healthcare). Further purification was carried out using a Superdex 75 10/300 GL column. Fractions containing the target protein were collected at low concentrations, frozen in liquid nitrogen, and stored at -80°C for functional study or concentrated to 10 mg/ml for crystallization.

### Crystallization, Data Collection, and Processing

Crystals of refolded BbtPGRP3 and insect cells expressed BbtPGRP3 (Gln19−Gly74, Ser87−Gly255) were grown at 289 K using the hanging drop vapor-diffusion method, by mixing 1 μl of protein solution with an equal volume of the reservoir solution (1.8 M Sodium Chloride, 0.1 M citric acid, pH 5.6 or 1.2 M Lithium sulfate, 0.1 M Tris-HCl, pH 8.5, respectively). Crystals were transferred to a cryoprotectant solution (reservoir solution supplemented with 30% glycerol) and flash-cooled with liquid nitrogen. The datasets of refolded BbtPGRP3 and insect cells expressed BbtPGRP3 were collected at a radiation wavelength of 0.9795 Å at the Shanghai Synchrotron Radiation Facility (Shanghai Institute of Applied Physics, Chinese Academy of Sciences) using the beamline BL17U at 100 K with a MX-225 CCD (Marresearch) and then processed with HKL2000 [53].

### Structure Determination and Refinement

The crystal structure of refolded PGRP domain was determined by molecular replacement using human PGRP-S (PDB code 1YCK, 51% of sequence identity with refolded PGRP domain) [54] as search models with MOLREP [55] in CCP4 [56]. Refinement was carried out using the maximum likelihood method implemented in REFMAC [57] and the interactive rebuilding process in Coot [58]. The structure of refolded PGRP domain was then used as search model to determine the structure of insect cells expressed BbtPGRP3. The structure of BbtPGRP3 was further refined with the refinement program from PHENIX [59] and rebuilt in COOT. The overall assessment of model quality was performed using MolProbity [60]. The data collection and structure refinement statistics of BbtPGRP3 and refolded PGRP domain were listed in Table 1. All structure figures were prepared with PyMOL [61].

**Table 1:** Data collection and refinement statistics.

	BbtPGRP3	Refolded PGRP domain
*Data collection*		
Space group	*P6*_*5*_	*P3*_*2*_ *21*
Unit cell		
* a*, *b*, *c* (Å)	77.206, 77.206, 82.607	79.596, 79.596, 82.262
* *α, β, γ (°)	90.00, 90.00, 120.00	90.00, 90.00, 120.00
Resolution range (Å)	50.00–2.70 (2.80–2.70) ^a^	50.00–2.80 (2.90–2.80)
Unique reflections	15,022 (1,511)	7,646 (740)
Completeness (%)	99.2 (99.2)	98.7 (98.5)
<*I/σ(I)*>	11.0 (2.9)	8.4 (3.1)
R_merge_ ^b^ (%)	10.1 (45.8)	16.2 (69.4)
Average redundancy	3.2 (3.2)	3.1 (3.2)
Wilson B factor (Å^2^)	33.3	48.0
*Structure refinement*		
Resolution range (Å)	50.00–2.70	50.00–2.80
R-factor^c^/R-free^d^ (%)	17.2/20.1	19.77/21.95
No. of atoms		
protein	1,691	1,252
Zinc	1	
water	74	19
Average B factors (Å^2^)		
protein	46.0	57.8
Zinc	44.9	
water	50.0	47.0
RMSD^e^ bond lengths (Å)	0.003	0.007
RMSD bond angles (°)	0.812	1.114
*Ramachandran plot* ^*f*^ *(residues*, *%)*		
Most favored (%)	97.75	96.93
Additional allowed (%)	2.25	3.07
Outliers (%)	0	0
PDB entry	4Z8I	4ZXM

^a^The values in parentheses refer to statistics in the highest bin.

^b^R_merge_ = ∑_hkl_∑_i_|I_i_(hkl)-<I(hkl)>|/∑_hkl_∑_i_I_i_(hkl), where I_i_(hkl) is the intensity of an observation and <I(hkl)> is the mean value for its unique reflection; Summations are over all reflections.

^c^R-factor = ∑_h_|Fo(h)-Fc(h)|/∑_h_Fo(h), where Fo and Fc are the observed and calculated structure-factor amplitudes, respectively.

^d^R-free was calculated with 5% of the data excluded from the refinement.

^e^Root-mean square-deviation from ideal values.

^f^Categories were defined by Molprobity.

### Determination of metal binding to the protein

The purified BbtPGRP3 in 20 mM Tris-HCl, pH 8.5, 150 mM NaCl was concentrated to 10 mg/ml and applied to the analyses. Briefly, 500 μl of protein sample was subjected to digestion by the aqueous method using the HNO_3_ and HClO_4_ (4:1, *v/v*) method. Afterwards, the digested sample was diluted with deionized water and analyzed by atomic absorption spectroscopy (Atomscan Advantage, Thermo Ash Jarell Corporation, USA).

### Computational docking

The docking of TCT or MPP to the PGRP domain of BbtPGRP3 was performed with HADDOCK server: the Expert interface [46, 62]. The initial coordinates of the PGRP domain, TCT, and MPP molecules were taken from the BbtPGPR3 crystal structure (PDB code 4Z8I), the complex structure of DmPGRP-LE-TCT (PDB code 2F2L) and HsPGlyRP3-MPP (PDB code 2APH). The predicted substrate binding residues in BbtPGRP3 (position number 122, 123, 156, 177, 178, 183, 230 and 238) together with the TCT or MPP molecule were used as active residues, whereas the residues neighboring the active residues were used as passive residues. The HADDOCK procedure was then performed with other parameters in default values. The HADDOCK score was used to rank the generated models. The lowest energy models were chosen.

### Chitin binding assays

Aliquots of 5 mg chitin beads (NEW ENGLAND BioLabs) were mixed with 5 μM of BbtPGRP3, the CBD, the PGRP domain and BbtPGRP3-W53A/W55A dissolved in 50 μl of 20 mM Tris-HCl, pH 8.5, 150 mM NaCl. The mixtures were incubated at 25°C for 2 h, and then centrifuged at 10,000 g for 10 min. The supernatants were treated with 5 × SDS sample buffer and used for analysis of unbound fraction. The pellets were washed three times with 200 μl 20 mM Tris-HCl, pH 8.5, 150 mM NaCl, and then boiled with 50 μl of 2 × SDS sample buffer. The unbound and bound samples were separated by 15% SDS-PAGE.

### Hydrolytic activity assays

The insoluble DAP-type and Lys-type PGNs were purified from *S*. *aureus* and *E*. *coli* independently, following the previously described protocols [63, 64]. The purified PGNs were lyophilized and then dyed with Remazol Brilliant Blue R (RBB, sigma) according to a previous report [65]. The dye release assay was routinely performed at 60°C with gentle shaking in 20 mM CAPS, pH 10.5, at a substrate concentration of about 8 mg/ml for 10 min. The reaction was stopped by addition of 1/2 vol 96% ethanol. The insoluble material was removed by centrifugation and the absorbance of the supernatant was measured at 595 nm. All experiments were performed three times.

### HPLC assays

MPP was kindly provided by Dr. David I Roper at the University of Warwick. The pentapeptide (PP) was synthesized by GL Biochem (Shanghai). The hydrolytic activity of BbtPGRP3 towards MPP was performed at 37°C for 8 h in a 45 μl reaction system containing MPP at a final concentration of 1 mM in 20 mM CAPS, pH 10.5. The reaction was initiated by adding purified BbtPGRP3 to a final concentration of 50 nM, and terminated by the addition of 5 μl 2% trifluoroacetic acid (TFA). After centrifugation at 12,000 g for 10 min, the supernatant was applied to a reversed-phase HPLC system (Agilent 1200 Series) which had been pre-equilibrated with the reaction buffer containing 0.05% TFA. A 0 to 15% acetonitrile gradient containing 0.05% TFA was used to elute the components from the C18 column (Eclipse XDS-C18 column, 4.6×150 mm; Agilent) at a flow rate of 1 ml/min. The eluted fractions were detected and quantitated by recording the absorbance at 220 nm.

### PGN binding assays

Aliquots of 1 mg purified DAP-type and Lys-type PGNs were mixed with 5 μM of BbPGRP3 dissolved in 50 μl of 20 mM Tris-HCl, pH 8.5, 150 mM NaCl and 5 mM EDTA. The mixtures were incubated at 25°C for 4 h, and then centrifuged at 10,000 g for 10 min. The supernatants were treated with 5 × SDS sample buffer and used for analysis of unbound fraction. The pellets were washed three times with 200 μl 20 mM Tris-HCl, pH 8.5, 150 mM NaCl, and then boiled with 50 μl of 2 × SDS sample buffer. The unbound and bound samples were separated by 15% SDS-PAGE.

## Supporting Information

S1 Fig HPLC spectra of MPP hydrolyzed by BbtPGRP3. The upper panel shows that the MPP was partially hydrolyzed to PP by BbtPGRP3. The two lower panels represent the peaks of MPP and PP standard samples. The eluted fractions were detected and quantitated by recording the absorbance at 220 nm. MPP, muramyl pentapeptide; PP, pentapeptide.
(TIF)

S2 Fig The binding affinity of BbtPGRP3 towards DAP-type or Lys-type PGNs. Purified BbtPGRP3 was incubated with insoluble DAP-type and Lys-type PGNs, respectively. Protein remaining in the supernatant (Unbound, U) and associated with the pellet (Bound, B) were analyzed by SDS-PAGE and Coomassie staining.
(TIF)

S3 Fig The hydrolytic activity of BbtPGRP3 towards DAP-type or Lys-type PGNs. Reactions containing 10 mg RBB-labeled *E*. *coli* or *S*. *aureus* PGNs were incubated with 50 nM BbtPGRP3. Undigested PGNs were pelleted by centrifugation and the absorbance of the supernatants at 595 nm were recorded. The results are expressed as mean values ± SD of three independent experiments.
(TIF)
