# Supplementary figures and images for: Activity Augmentation of Amphioxus Peptidoglycan Recognition Protein BbtPGRP3 via Fusion with a Chitin Binding Domain
Source: PLoS One. 2015 Oct 19;10(10):e0140953. doi: 10.1371/journal.pone.0140953 (PMC4610682; doi:10.1371/journal.pone.0140953)

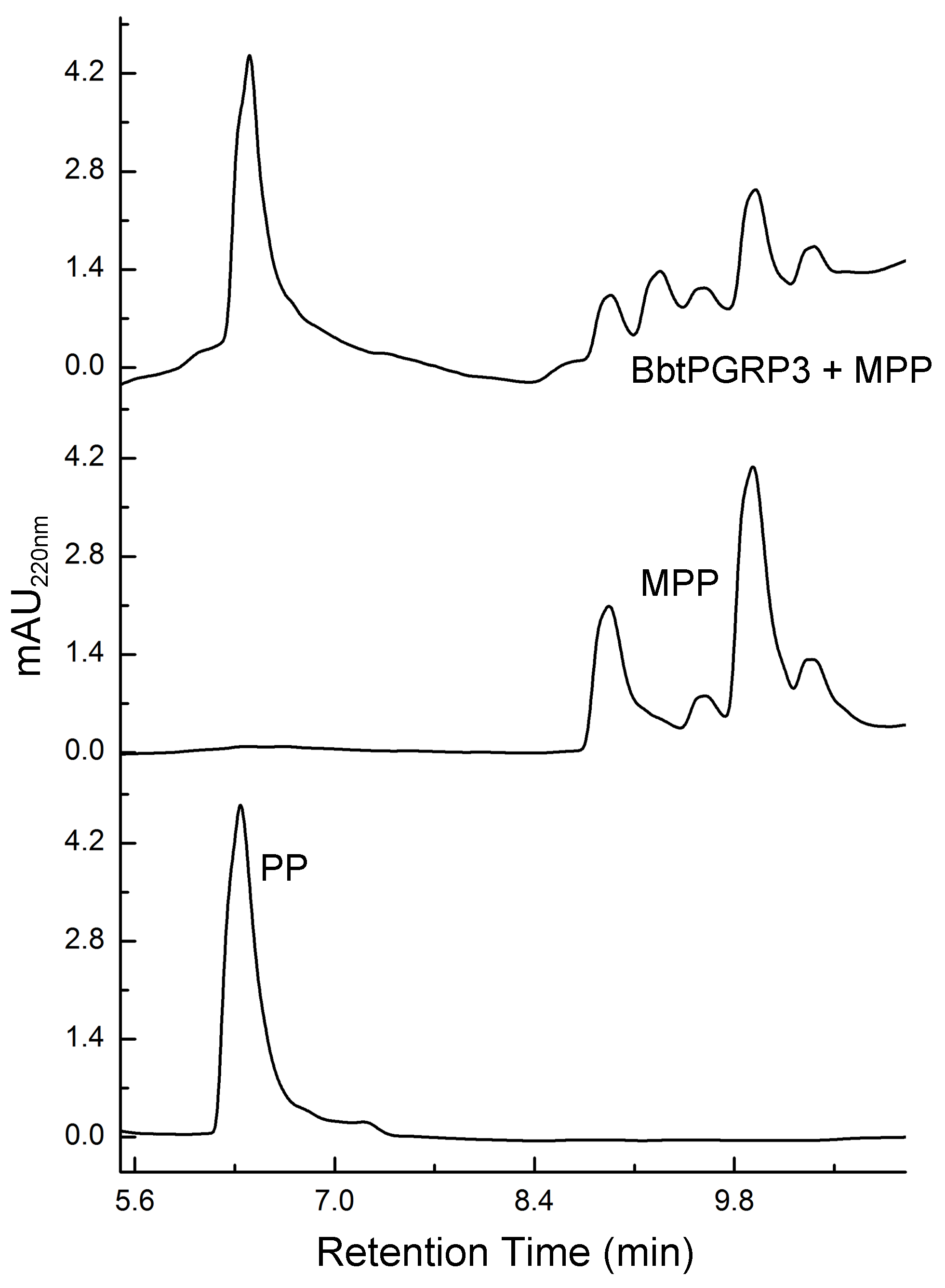

Supplement: S1 Fig — The upper panel shows that the MPP was partially hydrolyzed to PP by BbtPGRP3. The two lower panels represent the peaks of MPP and PP standard samples. The eluted fractions were detected and quantitated by recording the absorbance at 220 nm. MPP, muramyl pentapeptide; PP, pentapeptide. (TIF) [file pone.0140953.s001.tif]

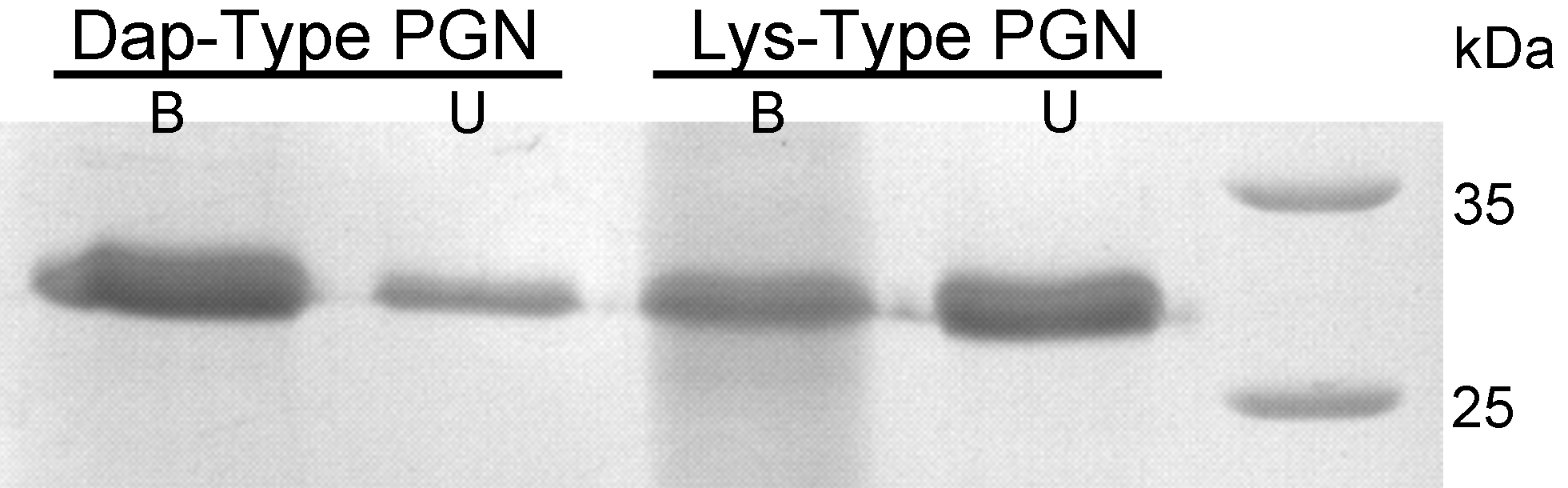

Supplement: S2 Fig — Purified BbtPGRP3 was incubated with insoluble DAP-type and Lys-type PGNs, respectively. Protein remaining in the supernatant (Unbound, U) and associated with the pellet (Bound, B) were analyzed by SDS-PAGE and Coomassie staining. (TIF) [file pone.0140953.s002.tif]

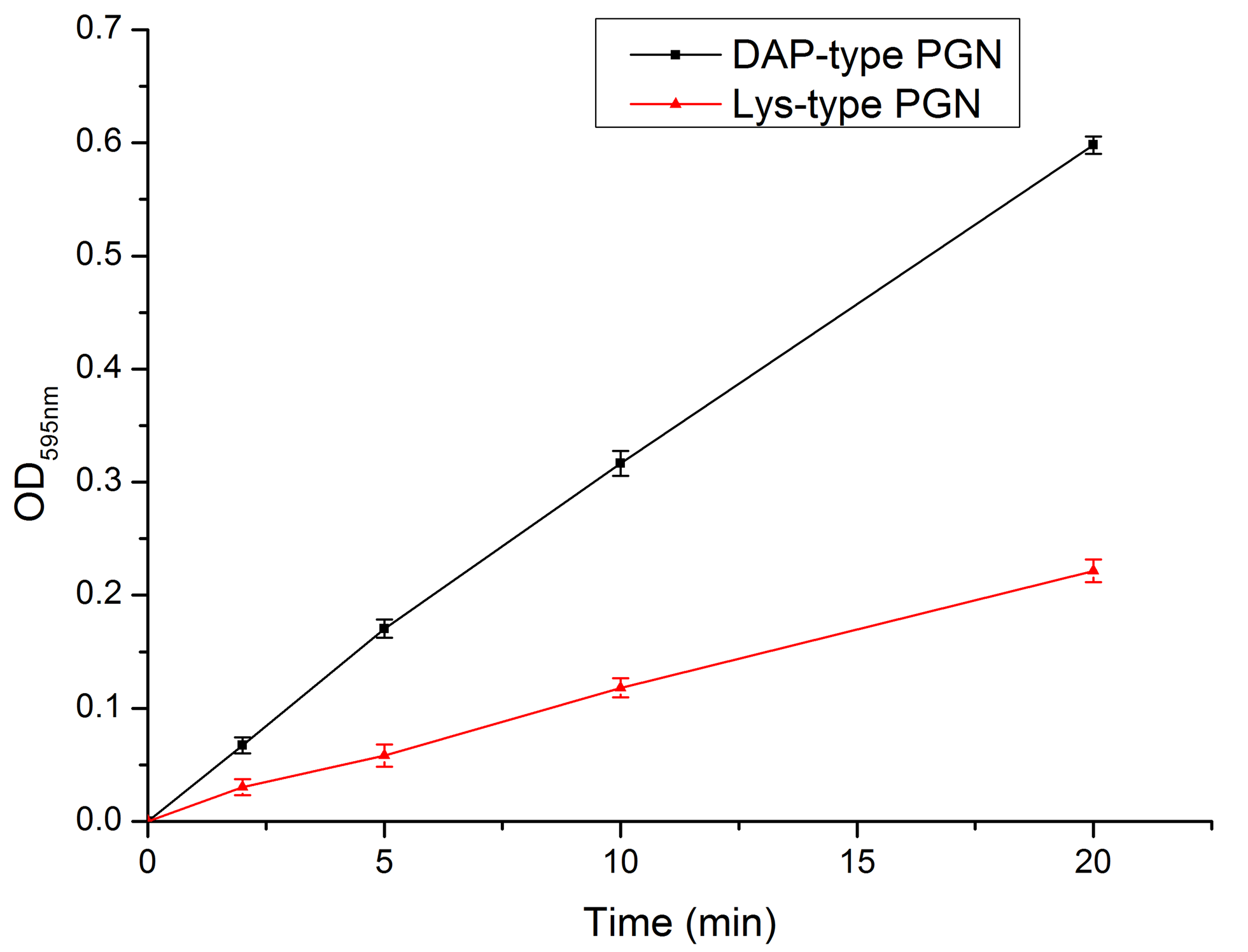

Supplement: S3 Fig — Reactions containing 10 mg RBB-labeled E. coli or S. aureus PGNs were incubated with 50 nM BbtPGRP3. Undigested PGNs were pelleted by centrifugation and the absorbance of the supernatants at 595 nm were recorded. The results are expressed as mean values ± SD of three independent experiments. (TIF) [file pone.0140953.s003.tif]
